# Adverse Reactions Manifesting as Restricted Eye Movement and Nasal Lesions in a 38-Year-Old Chinese Woman Following Cosmetic Hyaluronic Acid Injection: A Case Report

**DOI:** 10.7759/cureus.95139

**Published:** 2025-10-22

**Authors:** Nourah Alazemi, Saad Alsaleh, Hussain Al Hirmi, Ameen Al Awadhi

**Affiliations:** 1 Kuwait Institute for Medical Specializations (KIMS), Ministry of Health, Kuwait, KWT; 2 College of Medicine, Arabian Gulf University, Manama, BHR; 3 Department of Ophthalmology, Salmaniya Medical Complex, Manama, BHR; 4 Department of Dermatology, Salmaniya Medical Complex, Manama, BHR

**Keywords:** adverse filler reactions, cosmetic procedures, dermal filler, hyaluronic acid, nasal lesions, restricted eye movement, vascular occlusion

## Abstract

Hyaluronic acid (HA) is a widely used dermal filler in cosmetic procedures due to its biocompatibility and ability to provide volume and hydration to the skin. Despite its popularity and general safety, adverse reactions can occur, necessitating awareness and preparedness among clinicians.

This case report presents a 38-year-old Chinese woman who developed restricted eye movement likely affecting both horizontal and vertical movements. The specific muscles involved may include the medial and lateral rectus muscles, as well as the superior and inferior rectus and oblique muscles, contributing to the observed symptoms along with nasal lesions following an HA injection. Such complications are often attributed to inadvertent vascular occlusion, leading to ischemia in the affected areas.

Preventive measures and immediate treatment are crucial in managing these adverse effects. The administration of hyaluronidase, an enzyme that breaks down HA, is the primary intervention to mitigate the effects of vascular compromise. Additionally, aspirin may be prescribed to improve blood flow and reduce the risk of thrombosis.

In this case, prompt treatment with hyaluronidase and supportive therapy, including aspirin, led to significant improvement. Over several weeks, the patient experienced restored eye movement and resolution of nasal lesions.

## Introduction

Hyaluronic acid (HA) fillers are extensively utilized in cosmetic dermatology for facial enhancement due to their hydrating and volumizing properties. While generally considered safe, they can occasionally lead to severe complications, particularly when vascular compromise occurs [1,2]. This case report describes a 38-year-old Chinese woman who developed significant complications following HA filler injections, including skin lesions on the nose, restricted eye movements, dizziness, vomiting, and facial redness.

Vascular occlusion is a recognized risk associated with dermal fillers and can result in ischemic events and tissue necrosis if not promptly addressed [1,2]. Immediate intervention with hyaluronidase, which breaks down HA, is crucial to alleviate vascular obstruction and restore perfusion [2]. Additionally, aspirin may be prescribed to improve blood flow and reduce the risk of thrombosis [3].

This case report aims to underscore a rare but serious complication of HA injection, drawing attention to the key clinical signs, the value of early recognition, and the potential for favorable outcomes with timely management. Its significance lies in serving as a clinical reminder for practitioners to remain vigilant for such adverse events. In our patient, rapid intervention with hyaluronidase and supportive measures, including aspirin, resulted in marked improvement, with the gradual recovery of ocular motility and the complete resolution of nasal lesions over the following weeks.

## Case presentation

**Figure 1:**
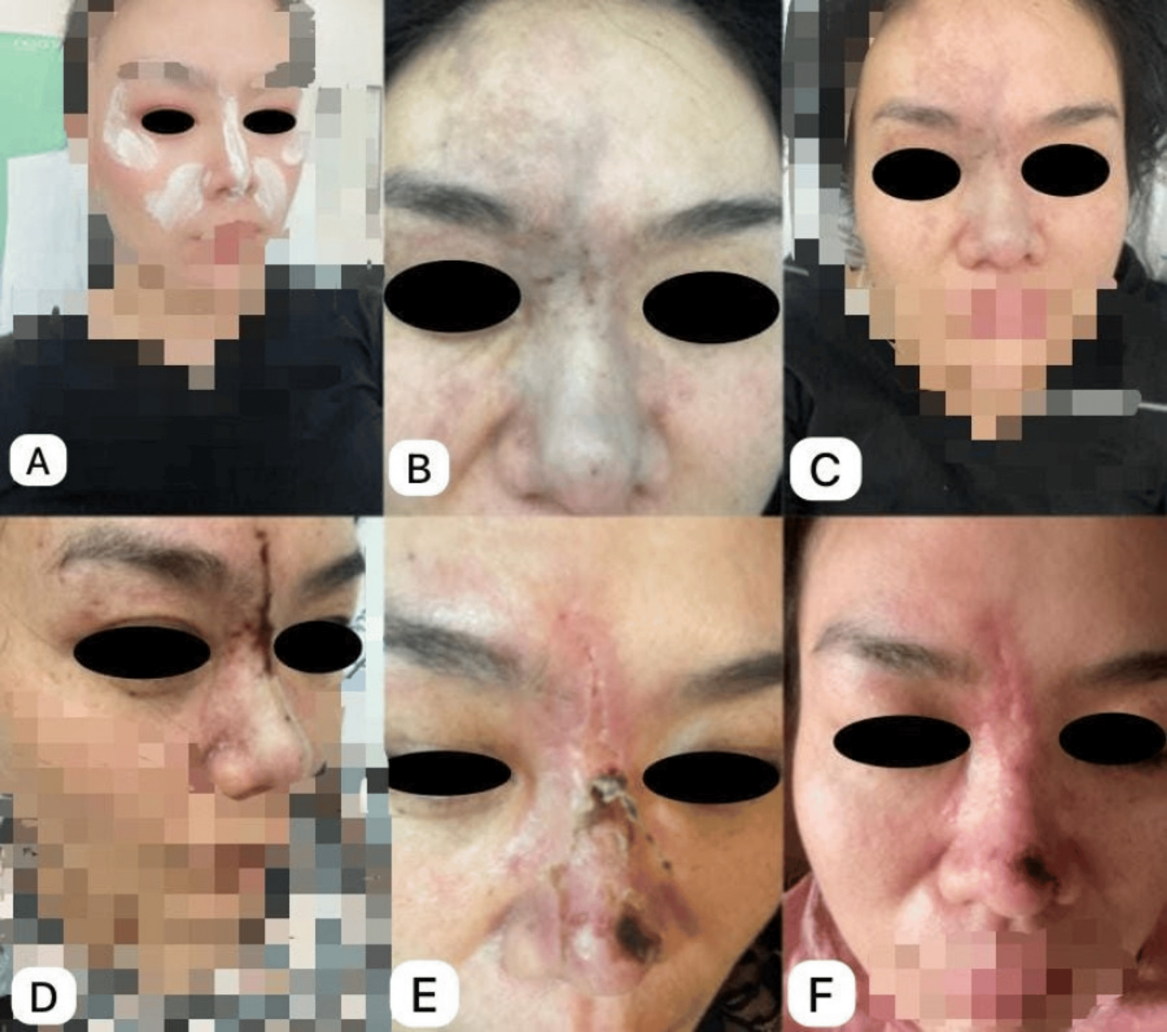
Presentation and stages of the resolution of the violaceous rash due to HA injection into the right nasolabial fold, depicting the progression from initial reaction through treatment and recovery stages for the 38-year-old Chinese woman (A) Pre-injection with HA baseline appearance. (B and C) Immediate post-injection reaction. (D and E) Stages of the violaceous rash, nasal crusting, red eye, and restricted eye movement. (F) Resolution after treatment with hyaluronidase, aspirin, and topical creams after one week The patient consented to have her identity revealed, and a written and signed consent statement was provided to the journal. HA: hyaluronic acid

The patient is a 38-year-old woman of Chinese descent who does not have any notable health issues or chronic conditions that could affect her medical treatment or diagnosis. This background suggests that she is generally healthy and has not previously experienced any major illnesses or surgeries that are relevant to her current situation. She chose to go to a private sector clinic for this procedure, seeking a more personalized and potentially specialized experience. She received an HA filler injection through a sharp needle into both nasolabial fold areas, potentially near the alar base of the nose. Figure 1A shows her appearance before the procedure. Shortly after the injection, within minutes, she experienced discomfort in the entire nasal region, particularly where the filler was injected into the nasolabial fold (Figure 1B-1C), which was partially alleviated by a non-steroidal anti-inflammatory drug, ibuprofen.

Twenty-seven hours post-injection, she developed bilateral restricted eye movements, involving both eyes, with limitation predominantly in upward and medial gaze. The affected extraocular muscles are most likely the superior rectus and medial rectus, with possible involvement of the inferior oblique. Ocular motility was restricted in nearly all directions, but especially in elevation (superiorly) and adduction (medially), as confirmed by orthoptic assessment. The restriction was most notable in upward and medial gaze, while lateral and inferior movements were comparatively less affected. This was accompanied by a violaceous rash and increasing redness around the left nasolabial fold (Figure 1D), raising concerns of vascular compromise involving the angular artery, a terminal branch of the facial artery, likely at the subcutaneous and dermal levels, due to the filler.

Further deterioration was observed, with noticeable crusting and worsening of the nasal lesions (Figure 1E), accompanied by significant restriction in eye movements, suggesting possible orbital involvement.** **Given these symptoms, she was clinically diagnosed with arterial compromise upon HA injection. CT angiography was performed, which was negative for retinal artery thrombosis but demonstrated decreased perfusion in the left angular artery and its branches along the left nasolabial fold and infraorbital region, consistent with localized vascular compromise.

The patient had no prior history of medication use and was otherwise healthy. The treatment plan involved aspirin and hyaluronidase injections of 350 units administered to the affected area every four hours over three days. The fixed dose and four-hour interval ensure the continuous enzymatic activity of hyaluronidase for effective HA breakdown and sustained tissue reperfusion.

After two weeks, the nasal lesions resolved completely, leaving only a few transient papules that gradually disappeared within an additional two weeks. Following this regimen, the patient showed significant improvement, including the complete resolution of ocular symptoms such as blurred vision and photophobia, as well as a marked reduction in cutaneous signs. After two weeks, the violaceous rash and erythema over the left nasolabial fold and infraorbital region resolved completely, leaving only a few transient papules, which gradually disappeared within an additional two weeks (Figure 1F).

## Discussion

This case report highlights the serious complications that can arise from HA filler injections, particularly the risk of vascular occlusion leading to restricted eye movement and nasal lesions. Although HA fillers are generally considered safe and are widely used in cosmetic procedures, especially in adults aged 21 years and older, this case emphasizes the need for clinician vigilance regarding potential adverse effects [3,4]. The complications observed in this patient are likely due to inadvertent intravascular injection into the angular artery, a terminal branch of the facial artery, or its adjacent branches. This led to localized ischemia affecting the medial rectus muscle, resulting in restricted ocular motility, as well as ischemic changes in the skin over the left nasolabial fold and infraorbital region, as shown in Figure 1D. This case underscores the importance of proper anatomical knowledge, slow injection techniques, and immediate recognition and treatment of vascular compromise to minimize the risk of long-term sequelae.

Vascular occlusion is a recognized risk associated with dermal fillers, which can lead to significant ischemic events and tissue necrosis if not promptly addressed [4]. The rapid onset of symptoms, including restricted eye movement and nasal lesions, emphasizes the need for a thorough understanding of facial anatomy and careful injection techniques to minimize risks (Figure 1B). The management of this case involved the timely administration of hyaluronidase, which is critical in reversing the effects of vascular compromise, particularly if initiated within the first 90 minutes to four hours of symptom onset. Hyaluronidase works by breaking down HA, alleviating pressure on occluded vessels and restoring blood flow. Additionally, the use of aspirin in the treatment regimen aimed to enhance circulation and reduce the risk of thrombosis, further supporting the management strategy [3]. The successful resolution of the patient's symptoms following these interventions reinforces the importance of early recognition and treatment of filler-related complications (Figure 1F).

This case contributes to the growing body of literature on HA filler safety and the necessity for ongoing education and preparedness among healthcare providers. It also highlights the potential complications associated with the use of hyaluronidase, such as localized allergic reactions, tissue swelling, or unintended diffusion leading to the degradation of native HA, underscoring the importance of precise dosing and administration. Clinicians performing cosmetic procedures must be aware of the potential for adverse reactions and be equipped with the knowledge and tools necessary to manage such events effectively. Continuous training and updates in best practices are essential for ensuring patient safety and improving outcomes in cosmetic dermatology.

## Conclusions

This case report emphasizes the potential complications that can arise from HA filler injections, specifically the risk of vascular occlusion leading to significant adverse effects. The development of restricted eye movement and nasal lesions in this patient underscores the importance of timely intervention and the necessity for clinicians to be prepared for such reactions. The effective use of hyaluronidase and supportive care contributed to the patient's significant improvement, reinforcing the value of early intervention strategies.

As the popularity of cosmetic procedures continues to grow, it is imperative for healthcare providers to remain vigilant and informed about the risks associated with HA fillers. This case highlights the need for comprehensive patient education regarding potential complications and the importance of maintaining a high level of preparedness to ensure patient safety and optimal outcomes. 

Clinicians must have hyaluronidase readily available and be properly trained to manage vascular complications that may arise during filler treatments.
